# Supplementary material for: Microenvironmental IL1β promotes breast cancer metastatic colonisation in the bone via activation of Wnt signalling
Source: Nat Commun. 2019 Nov 1;10:5016. doi: 10.1038/s41467-019-12807-0 (PMC6825219; doi:10.1038/s41467-019-12807-0)
Supplement: Supplementary file 1 — Supplementary Information [file 41467_2019_12807_MOESM1_ESM.pdf]

Microenvironmental IL1β promotes breast cancer metastatic colonisation in the bone via activation of Wnt signalling

Eyre et al 2019

Supplementary Information

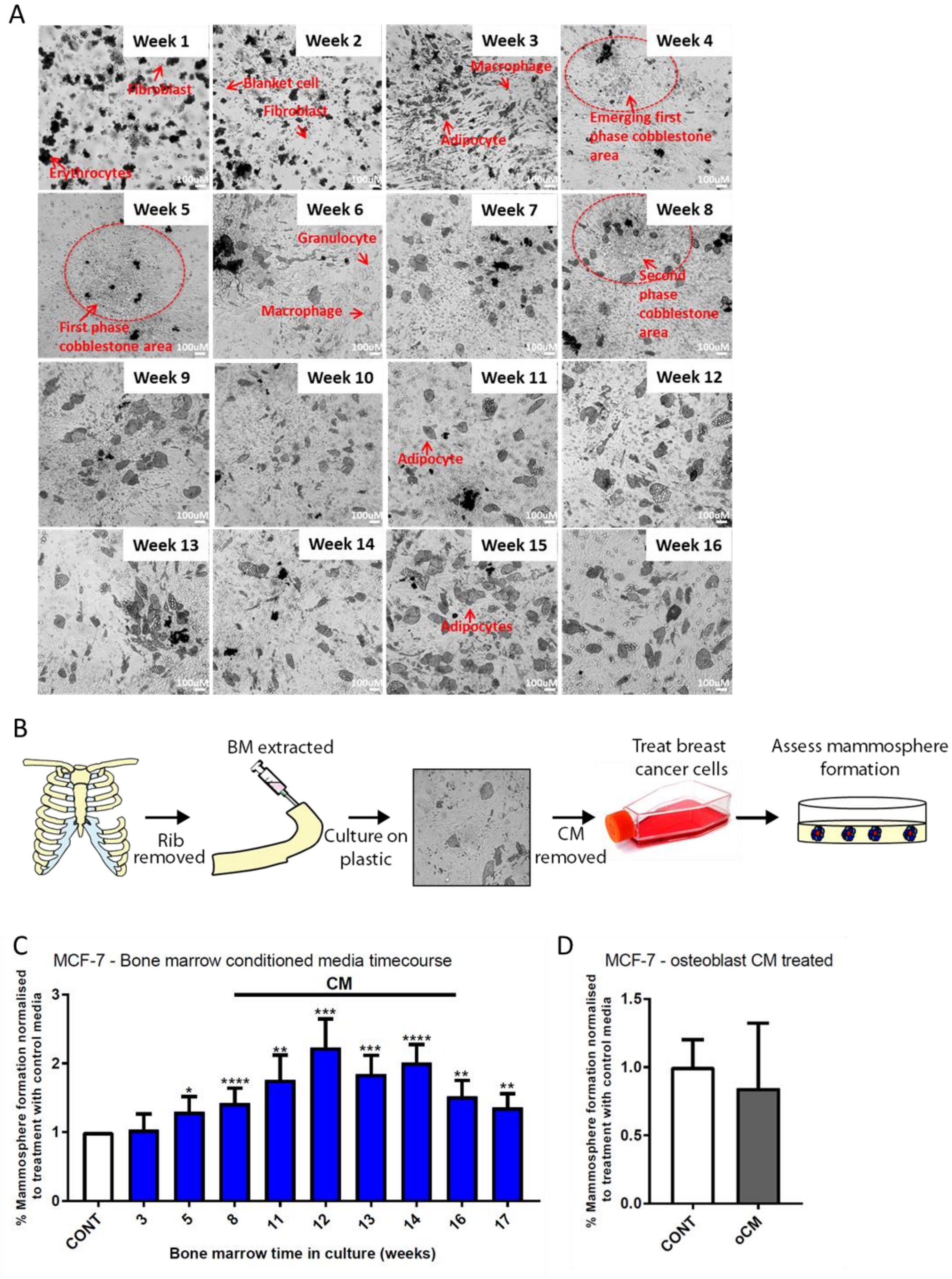

Supplementary Figure 1: Bone marrow derived factors promote breast CSC colony formation.
(A) Bone marrow growth images. Bone marrow was extracted from patient rib bones and cultured continuously on plastic. This is a dynamic culture system, with bone marrow cell composition changing over time. In the immediate stages following extraction (Week 1) large clumps of

erythrocytes are seen, these gradually die as the culture progresses. At Week 2 of culture fibroblasts develop, and haematopoietic progenitor cells home to these. By Week 4 bone marrow stroma is intact and the bone marrow undergoes a first phase of haematopoiesis from late haematopoietic cells. This is demonstrated by cobblestone areas, which in turn produce macrophages and granulocytes (Week 6). At Week 8 a second phase of haematopoiesis, from early progenitor cells, takes place. This produces granulocyte-macrophage progenitors, which secrete growth factors into the media. Haematopoietic cobblestone areas are maintained until approximately Week 11, with their growth supplemented by adipocytes. From Week 11 cobblestone areas begin to break down, and by Week 14 the culture is dominated by stroma, macrophages and adipocytes. Scale bar 100µM. (B) Workflow schematic. Rib bones were removed from patients undergoing access surgery for non-malignant renal failure. Bone marrow was extracted and cultured on plastic without passage for 20 weeks, with conditioned media (CM) removed weekly. Breast cancer cells in culture were treated with CM from bone marrow, and CSC colony forming activity assessed by mammosphere formation. (C) Conditioned media taken from bone marrow cultured for 3 weeks did not stimulate mammosphere colony formation in MCF-7 cells, whereas conditioned media from bone marrow cultured from 5-17 weeks significantly stimulated mammosphere formation. The largest increase in mammosphere formation was seen with conditioned media from weeks 8-16, therefore this was used for future experiments. Data is presented as fold change in percentage mammosphere formation following treatment with CM, normalised to treatment with control media for each time point. (D) Conditioned media from the osteoblast cell line hFOB1.19 (oCM) did not stimulate mammosphere colony formation in MCF7 cells. Data is presented as percentage mammosphere formation normalised to treatment with control media. All graphs represent mean±SEM, *p<0.05, **p<0.01, ***p<0.001, ****p<0.0001.

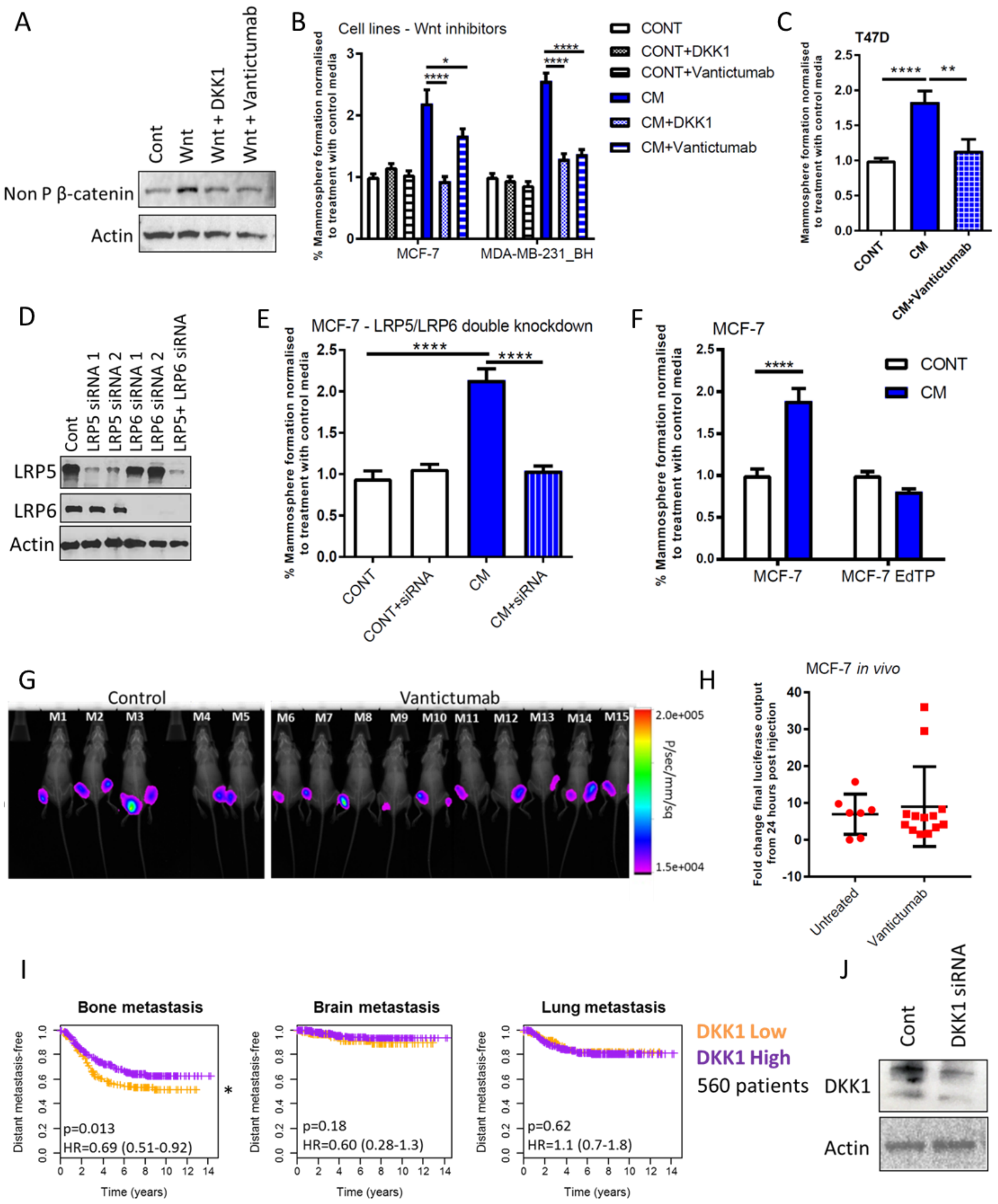

Supplementary Figure 2: CSC colony formation in response to bone marrow secreted factors is mediated by Wnt signalling. (A) 4 hours treatment with 100nm Wnt3A increased active β-catenin protein expression in 293T cells. This could be reversed by treatment with Wnt inhibitors DKK1 (50ng/ml) and Vantictumab (50µg/ml). (B) Inhibition of Wnt signalling with 50ng/ml DKK1 or

50µg/ml Vantictumab reversed the induction of mammosphere colony formation by bone marrow CM in MCF-7 and MDA-MV-231_BH cell lines. Inhibition of Wnt signalling did not reduce mammosphere formation in cells treated with control media. (C) Inhibition of Wnt signalling with 50µg/ml Vantictumab reversed the induction of mammosphere colony formation by bone marrow CM in the T47D cell line. (D) The LRP5 and LRP6 receptors could be knocked down specifically both alone and in combination following treatment with 25nM siRNA for 72 hours (combination shown is LRP5 siRNA1 and LRP6 siRNA 2). (E) Combination knockdown of LRP5 and LRP6 in MCF-7 cells prior to treatment with CM abrogated the effect of CM on mammosphere formation. (F) MCF-7 cells with a dominant negative TCF7 rendered incapable of Wnt signalling (MCF-7 EdTP) did not respond to CM with increased mammosphere formation. (G) Tumours cells were present in 7/10 (70%) of femurs in control mice and 13/20 (65%, no significant difference) of Vantictumab treated mice 24 hours following intra-femoral injection of $1x10^5$ MCF-7 cells. (H) Luciferase signal output in control and Vantictumab treated mice at experiment termination was not significantly different. Individual data points represent individual femurs. (I) Survival analysis based on DKK1 expression (absent/marginal (orange) vs present (purple)) from 3 published datasets (EMC192, EMC286, MSK82). Absent/marginal DKK1 expression in primary breast cancers was associated with poor outcomes in patients with bone metastasis, but not lung or brain metastases. HR; Hazard ratio. (J) DKK1 protein could be knocked down in MDA-MB-231 cells for 72 hours using 25nM siRNA. All graphs represent mean±SEM.*p<0.05, **p<0.01, ***p<0.001, ****p<0.0001.

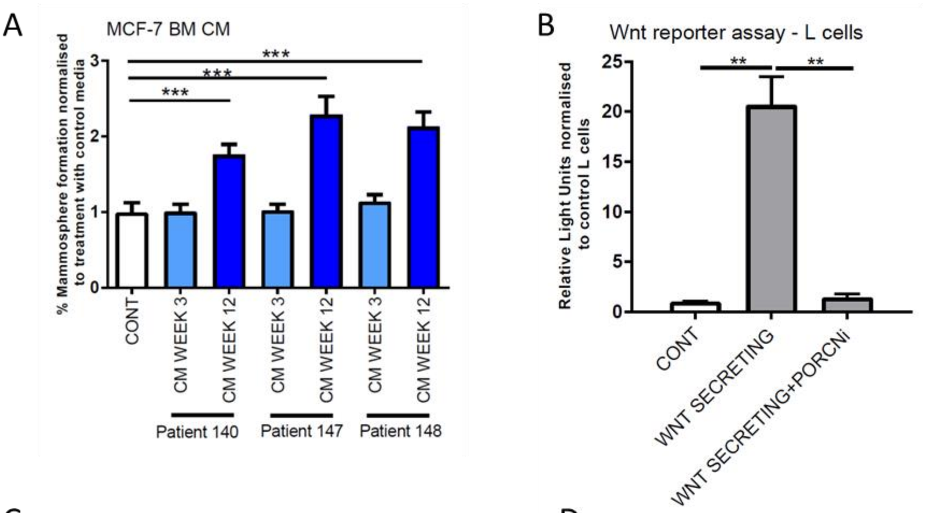

C

Cytokine	Fold change from non-stimulatory CM to stimulatory CM
IL-15	12.65
IL-1β	9.48
GCSF	4.36
IL-13	2.34
IL-5	2.12
FGF-4	2.04
Oncostatin M	2.01
NT-4	1.83
IL-6	1.73
MIP-1delta	1.67

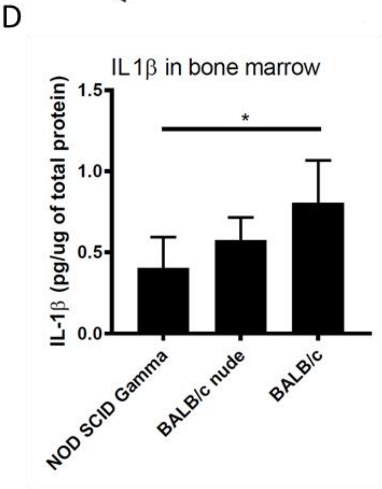

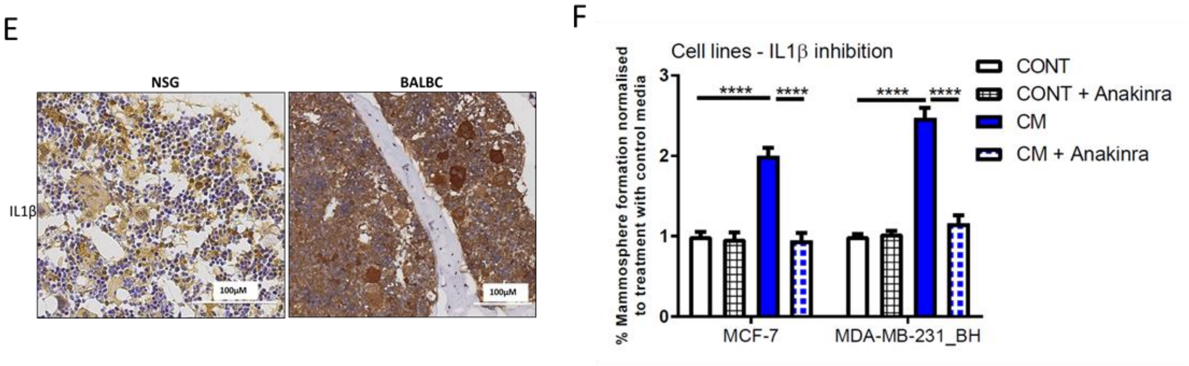

Supplementary Figure 3: Bone marrow derived IL1β promotes Wnt signalling and CSC colony formation. (A) Week 12 but not Week 3 conditioned media from bone marrow used for gene expression arrays stimulated mammosphere formation in MCF7 cells. Mammosphere formation of MCF7 cells following treatment with week 3 and week 12 CM from patients 140, 147 and 148 is shown. (B) Treatment with 100μM of the porcupine inhibitor LGK974 inhibited Wnt signalling from Wnt3A secreting L-cells, as measured by TCF4-driven reporter assay. (C) Fold change in expression

of the top 10 cytokines increased in stimulatory (week 8) CM from non-stimulatory (week 3) CM, as determined by Cytokine Array analysis. (D) ELISA analysis of bone marrow from immune competent (BALB/c) and immune deficient (BALB/c Nude, NOD SCID Gamma (NSG)) mice (n=6-8 per group). Data shown are mean +/- SD of IL-1β concentrations and are normalised to ug of total protein. (E) Immunohistochemical staining for IL1β in mouse bone sections in immune competent (BALB/c) and immune deficient (NSG) mice. Scale bar 100µM. (F) Treatment with Anakinra significantly reduced mammosphere CSC colony formation in MCF-7 and MDA-MB-231_BH cells in response to CM. Anakinra did not reduce CSC colony formation in cells treated with control media. All graphs represent mean±SEM, *$p<0.05$, **$p<0.01$, ***$p<0.001$, ****$p<0.0001$.

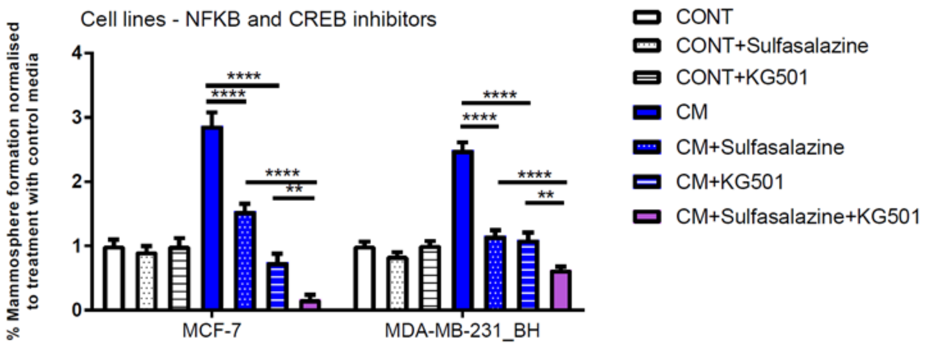

Supplementary Figure 4: IL1β promotes Wnt signalling in breast cancer cells via an induction in NFKB and CREB. Inhibiting NFKB using 5mM of sulfasalazine or inhibiting CREB using 10µM of KG501 reversed the induction in mammosphere formation by CM in MCF-7 and MDA-MB-231_BH cell lines. Sulfasalazine and KG-501 treatment did not reduce mammosphere formation in cells treated with control media. Combined inhibition with Sulfasalazine and KG501 gave further reduction in mammosphere formation than either treatment alone. All graphs represent mean±SEM, *$p<0.05$, **$p<0.01$, ***$p<0.001$, ****$p<0.0001$.

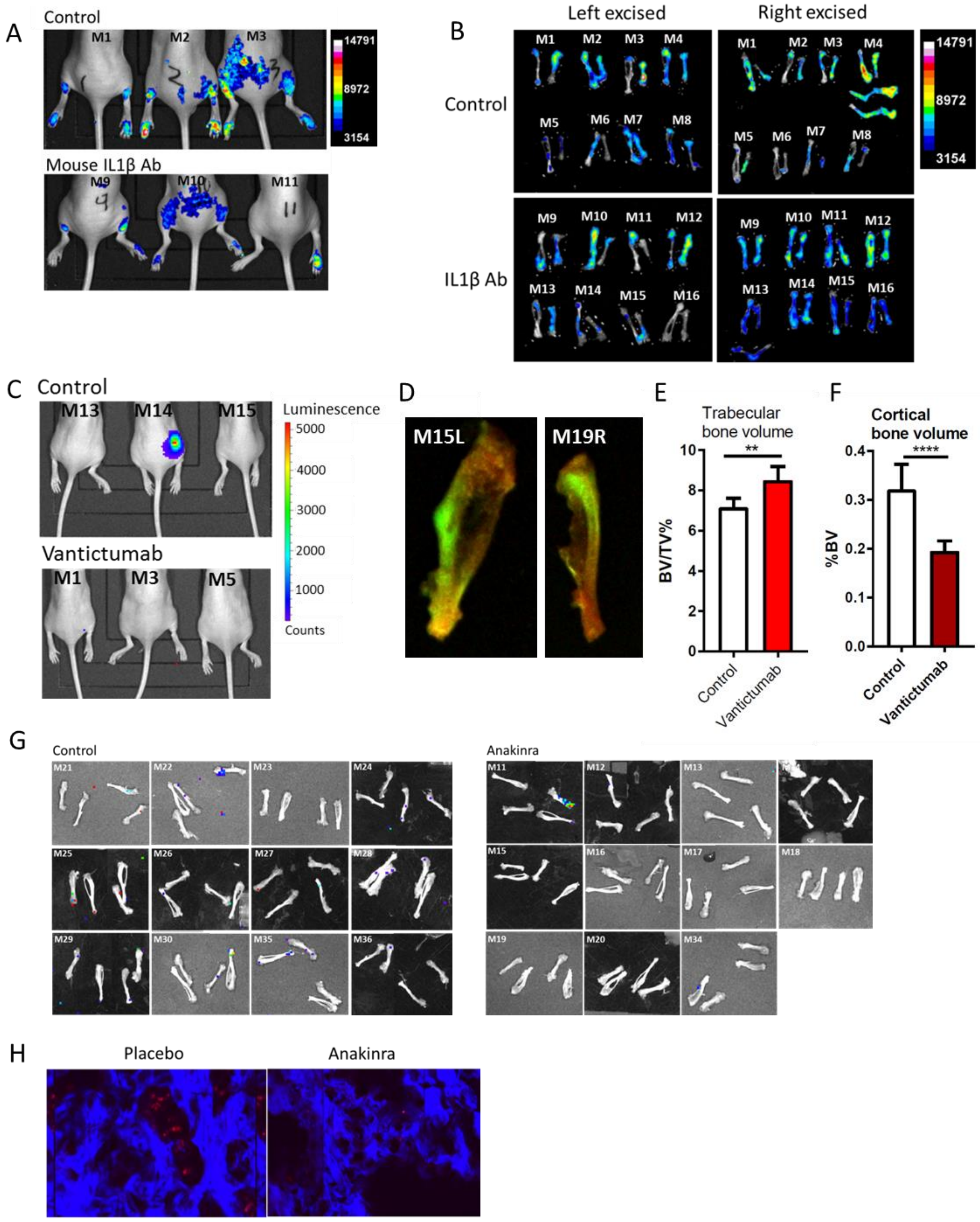

Supplementary Figure 5: Systemic inhibition of either IL1β or Wnt signalling prevents bone metastasis *in vivo*. (A) IL1β was inhibited systemically in a spontaneous MDA-MB-231_BH bone metastatic mouse model with 2mg/kg anti-IL1β antibody for 30 days. Tumour cells were visualised by IVIS in vivo imaging prior to termination. Images show example tumours in hind limbs mice in control (M1-M3) vs. IL1β antibody treated (M9-M11) mice after 30 days. Note autofluorescence is observed in mice feet. (B) IVIS images showing excised hind limbs of control vs. IL1β antibody

treated mice at experiment termination. (C) Wnt signalling was inhibited systemically in the MDA-MB-231_BH bone metastatic model with 15mg/kg Vantictumab for 30 days. Tumour cells were visualised by IVIS in vivo imaging prior to termination. Images show example tumours in hind limbs mice in control (M13-M15) vs. Vantictumab treated (M1, M3, M5) mice after 30 days. (D) Light tools images showing additional tumours identified by GFP expression in excised hind limbs at experiment termination (M15R and M19L). (E) Quantification of trabecular bone volume in control and Vantictumab treated mice. Data is presented as percentage bone volume/total volume (%BV/TV) with 5 femurs scored in the control group and 7 in the Vantictumab treated group. (F) Quantification of cortical bone volume in control and Vantictumab treated mice. Data is presented as % bone volume (%BV) with 6 femurs scored in the control group and 7 in the Vantictumab treated group. (G) IVIS images showing excised hind limbs of control vs. Anakinra treated mice at PDX experiment termination. (H) Two-photon images showing immunohistochemical staining of disseminated tumour cells in placebo and Anakinra treated mice without overt metastases of phycoerythrin conjugated cytokeratin 19 (sc376126) (red) in bone (blue). All graphs represent mean±SEM, *p<0.05, **p<0.01, ***p<0.001, ****p<0.0001.

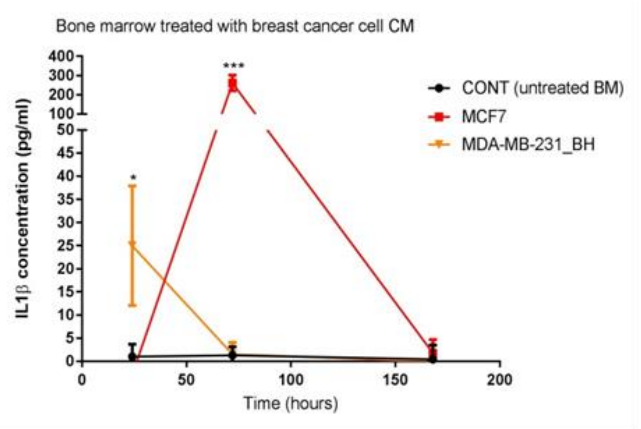

Supplementary Figure 6: Tumour cell conditioned media promotes IL1β secretion by the bone marrow. Bone marrow samples growing in culture were treated with either conditioned media from MCF7 cells, conditioned media from MDA-MB-231_BH cells, or control media. IL1β levels were assessed in these cultures by ELISA at 24 hours, 72 hours, and 7 days after treatment. Increases in IL1β levels were seen following either 72 hours of treatment with MCF7 cell conditioned media (red line; $p=0.0004$), or 24 hours with MDA-MB-231_BH conditioned media (orange line, $p=0.0306$). *$p<0.05$, **$p<0.01$, ***$p<0.001$, ****$p<0.0001$.

Patient ID	Gender	Age
GU114	Female	65
GU116	Male	48
GU120	Male	Young
GU124	Female	74
GU129	Male	39
GU140	Male	NA
GU147	Male	61
GU148	Male	54
GU165	Male	76
GU175	Female	71
GU176	Male	69
GU177	NA	NA

Supplementary Table 1: Characteristics of bone marrow donors used in this study. Gender and age are presented for each patient where CM was taken from bone marrow in culture. NA; Data not available.

Patient	Type	Grade	ER	PR	Her2	Neoadjuvant Treatment
BB6RC25	ILC	2	Pos	Pos	Neg	None
BB6RC28	IDC/ DCIS	3	Neg	Neg	Neg	None
BB6RC52	IDC	3	Neg	Neg	Neg	None
BB6RC53	IDC	2	Pos	Pos	Neg	Letrozole
BB6RC64	ILC	2	Pos	Neg	Neg	Arimidex
BB6RC66	IDC	2	Pos	Neg	Neg	None
BB6RC72	IDC	3	Neg	Neg	Neg	None
BB6RC80	IDC	2	Pos	Pos	Neg	None
BB6RC86	IDC	3	Pos	Pos	Neg	None
BB6RC87	IDC/ DCIS	3	Pos	Neg	Pos	None
BB6RC88	IDC	3	Neg	Pos	Neg	None
BB6RC94	IDC	3	Neg	Neg	Neg	None
BB6RC100	IDC	3	Neg	Neg	Pos	None
BB6RC114	IDC	2	Neg	Neg	Neg	None
BB6RC136	IDC	3	Neg	Neg	Neg	None
BB6RC139	IDC	1	Pos	Pos	Neg	None
BB6RC141	Fung. IMC	NA	Neg	Neg	Neg	FEC-T
BB6RC160	IDC	3	Pos	Neg	Pos	Arimidex
BB6RC174	IDC	2	Pos	Pos	Neg	Tamoxifen
BB7029	IDC	3	Pos	Pos	Pos	None
BB7030	IDC	2	Pos	Pos	Neg	None
BB7031	IDC	1	Pos	Pos	Neg	None
BB7047	IDC	2	Pos	Pos	Neg	None
BB7056	IDC	2	Pos	Pos	Neg	None

Supplementary Table 2: Characteristics of early patient-derived tumours used in the study. Breast cancer type, grade, hormone receptor status and neo-adjuvant therapy is presented for each patient included in this study. ILC; invasive lobular carcinoma, IDC; invasive ductal carcinoma, DCIS; Ductal carcinoma in situ, Fung. IMC; fungating invasive mucinous carcinoma, ER; oestrogen receptor, PR; progesterone receptor, Her2; Her2 receptor, Pos; Positive, Neg; Negative, NA; data not available. All patients with Her2 scores of 2+ were confirmed negative by FISH analysis.

Patient ID	Sample Type	ER	PR	Her2
BB3RC71	PE	Pos	Pos	Pos
BB3RC79	PE	Neg	Neg	Neg
BB7007	Asc	Pos	Pos	Neg
BB7034	PE	Neg	Pos	Pos

Supplementary Table 3. Characteristics of metastatic samples used in this study. Sample type and hormone receptor status are presented for each patient included in this study. PE; pleural effusion, Asc; Ascitic fluid, ER; oestrogen receptor, PR; progesterone receptor, Her2; Her2 receptor, Pos; Positive, Neg; Negative.
