# Supplementary material for: Microenvironmental IL1β promotes breast cancer metastatic colonisation in the bone via activation of Wnt signalling
Source: Nat Commun. 2019 Nov 1;10:5016. doi: 10.1038/s41467-019-12807-0 (PMC6825219; doi:10.1038/s41467-019-12807-0)
Supplement: Supplementary file 3 — Reporting Summary [file 41467_2019_12807_MOESM3_ESM.pdf]

nature research

Corresponding author(s): Rob Clarke
Penelope Ottewell

☒ Initial submission ☐ Revised version ☐ Final submission

Life Sciences Reporting Summary

Nature Research wishes to improve the reproducibility of the work that we publish. This form is intended for publication with all accepted life science papers and provides structure for consistency and transparency in reporting. Every life science submission will use this form; some list items might not apply to an individual manuscript, but all fields must be completed for clarity.

For further information on the points included in this form, see Reporting Life Sciences Research. For further information on Nature Research policies, including our data availability policy, see Authors & Referees and the Editorial Policy Checklist.

Experimental design

1. Sample size

Describe how sample size was determined.	For all animal studies, experiments were designed with the advice of a University of Manchester statistician who provided support to determine the optimal multi-variance analysis which could be performed, thereby reducing and optimising the number and size of groups using observations from experiments performed in the lead up to these proposed studies. Using 80% power and 95% confidence, 25% practical difference and 40% coefficient of variation, group sizes of 6 animals were recommended as sufficient. However, we were aware from pilot experiments that an average of one mouse per group is lost following injection (for example due to not recovering from anaesthetic). Therefore we elected to use 8 mice/group to overcome this.

2. Data exclusions

Describe any data exclusions.	No data were excluded from analyses.

3. Replication

Describe whether the experimental findings were reliably reproduced.	Most experiments were replicated several times with reproducible results, as indicated in figure legends. For all graphs, individual data points are shown to demonstrate experiment variability.

4. Randomization

Describe how samples/organisms/participants were allocated into experimental groups.	Randomisation of mice into treatment groups was not required, all mice used were the same age and sex.

5. Blinding

Describe whether the investigators were blinded to group allocation during data collection and/or analysis.	Investigators were not blinded to group allocation during data collection.

Note: all studies involving animals and/or human research participants must disclose whether blinding and randomization were used.

6. Statistical parameters

For all figures and tables that use statistical methods, confirm that the following items are present in relevant figure legends (or in the Methods section if additional space is needed).

n/a	Confirmed	
☐	☒	The exact sample size (*n*) for each experimental group/condition, given as a discrete number and unit of measurement (animals, litters, cultures, etc.)
☐	☒	A description of how samples were collected, noting whether measurements were taken from distinct samples or whether the same sample was measured repeatedly
☐	☒	A statement indicating how many times each experiment was replicated
☐	☒	The statistical test(s) used and whether they are one- or two-sided (note: only common tests should be described solely by name; more complex techniques should be described in the Methods section)
☒	☐	A description of any assumptions or corrections, such as an adjustment for multiple comparisons
☐	☒	The test results (e.g. *P* values) given as exact values whenever possible and with confidence intervals noted
☐	☒	A clear description of statistics including central tendency (e.g. median, mean) and variation (e.g. standard deviation, interquartile range)
☐	☒	Clearly defined error bars

See the web collection on statistics for biologists for further resources and guidance.

Software

Policy information about availability of computer code

7. Software

Describe the software used to analyze the data in this study.	GraphPad Prism 7

For manuscripts utilizing custom algorithms or software that are central to the paper but not yet described in the published literature, software must be made available to editors and reviewers upon request. We strongly encourage code deposition in a community repository (e.g. GitHub). *Nature Methods* guidance for providing algorithms and software for publication provides further information on this topic.

Materials and reagents

Policy information about availability of materials

8. Materials availability

Indicate whether there are restrictions on availability of unique materials or if these materials are only available for distribution by a for-profit company.	Patient Derived Xenograft tumours are available upon MTA.

9. Antibodies

Describe the antibodies used and how they were validated for use in the system under study (i.e. assay and species).	Antibodies used were as follows: LRP5 (Cell signalling 3889), LRP6 (Abcam ab75358), Non-phospho active β-catenin (Cell signalling 8814), DKK1 (Cell Signalling 4687), NFKB p65 (8242 P, Cell Signalling), phospho-CREB Ser133 (9198S), IL-1β (ab9722).

10. Eukaryotic cell lines

a. State the source of each eukaryotic cell line used.	Cell lines were purchased from ATCC or from internal collaboration within the University of Manchester.
b. Describe the method of cell line authentication used.	Cell lines were authenticated by multiplex PCR assay using the AmpF/STR system (Life Technologies, Paisley, UK).
c. Report whether the cell lines were tested for mycoplasma contamination.	All cell lines were confirmed negative for mycoplasma contamination on a monthly basis.
d. If any of the cell lines used are listed in the database of commonly misidentified cell lines maintained by ICLAC, provide a scientific rationale for their use.	N/A

Animals and human research participants

Policy information about studies involving animals; when reporting animal research, follow the ARRIVE guidelines

11. Description of research animals

Provide details on animals and/or animal-derived materials used in the study.	For intra-femoral and subcutaneous injections, 6 week old NSG (NOD.Cg-Prkdcscid Il2rgtm1Wjl/SzJ) were used. For spontaneous bone metastases experiments, 8 week old female BALB/c Nude mice were used.

Policy information about studies involving human research participants

12. Description of human research participants

Describe the covariate-relevant population characteristics of the human research participants.	Relevant information on human participants is provided in Extended Data Tables 1-3.
