# Supplementary material for: Microenvironmental IL1β promotes breast cancer metastatic colonisation in the bone via activation of Wnt signalling
Source: Nat Commun. 2019 Nov 1;10:5016. doi: 10.1038/s41467-019-12807-0 (PMC6825219; doi:10.1038/s41467-019-12807-0)

Microenvironmental IL1β promotes breast cancer metastatic colonisation in the bone via activation of Wnt signalling

Eyre et al 2019

Source Data

MDA-MB-231_BH

NFKB

Lane 1 Control, Lane 2 IL1β treated

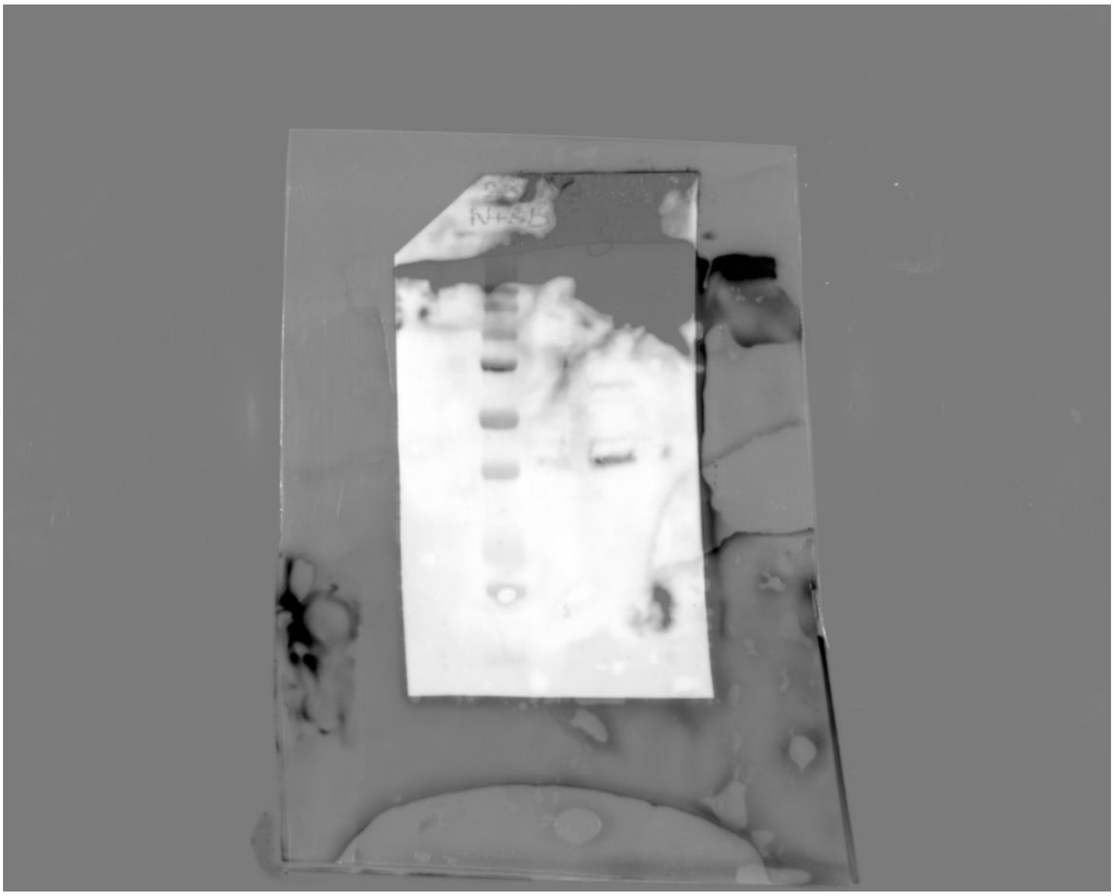

MDA-MB-231_BH

Lamin

Lane 1: Control, Lane 2 IL1β treated

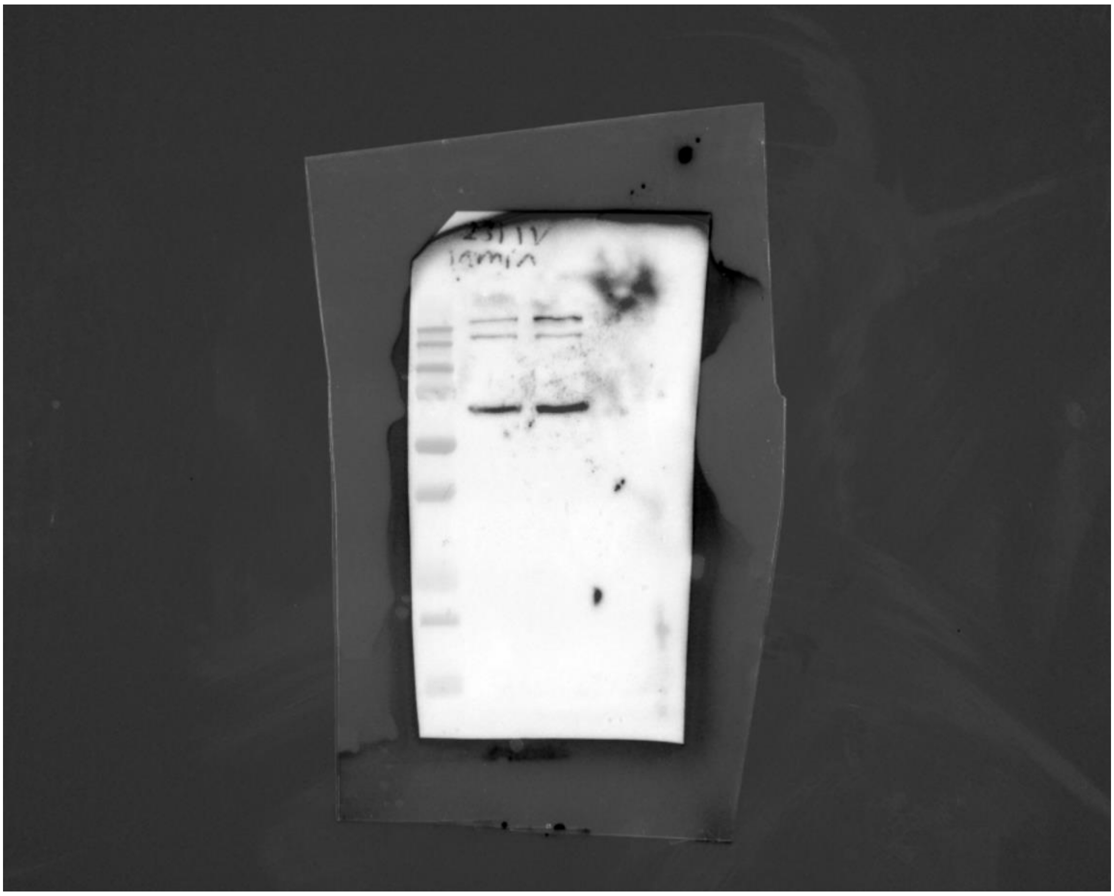

MCF7

NKFB

Lane 1: Control, Lane 2: IL1β treated

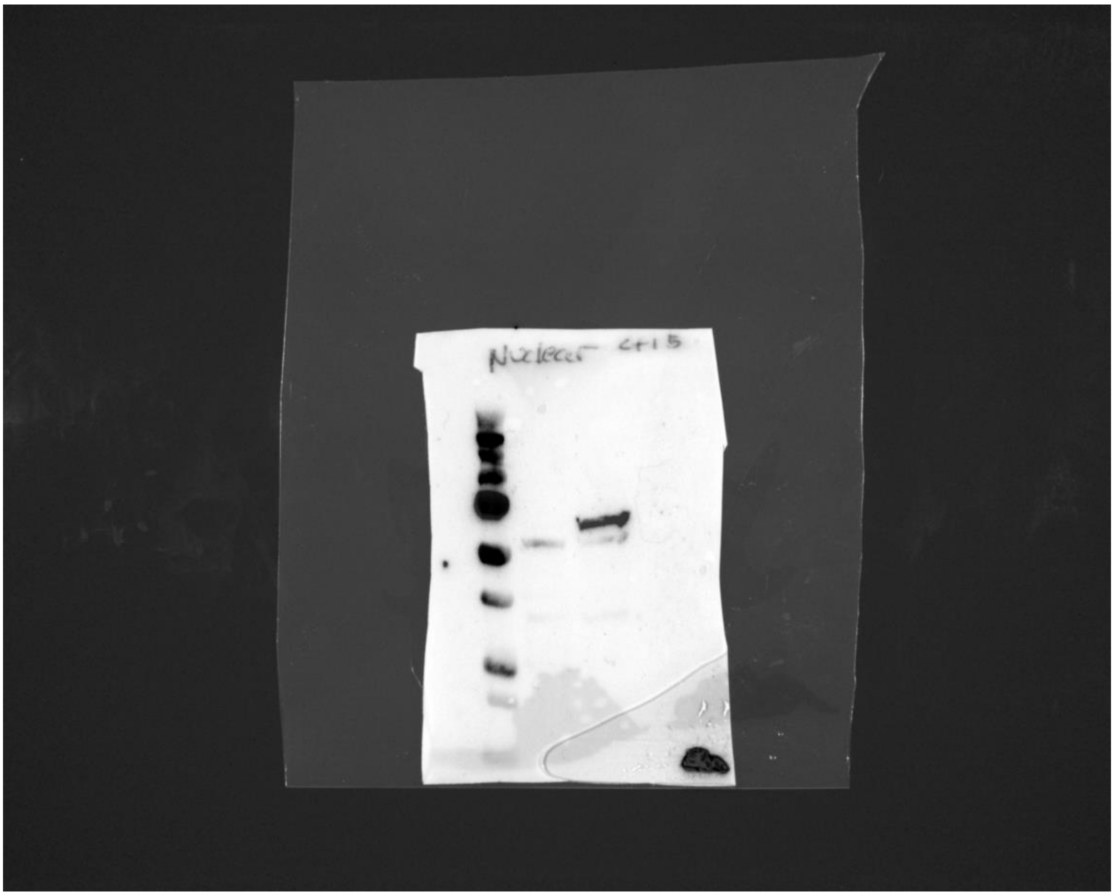

MCF7
Lamin
Lane 1: Control, Lane 2: IL1β treated

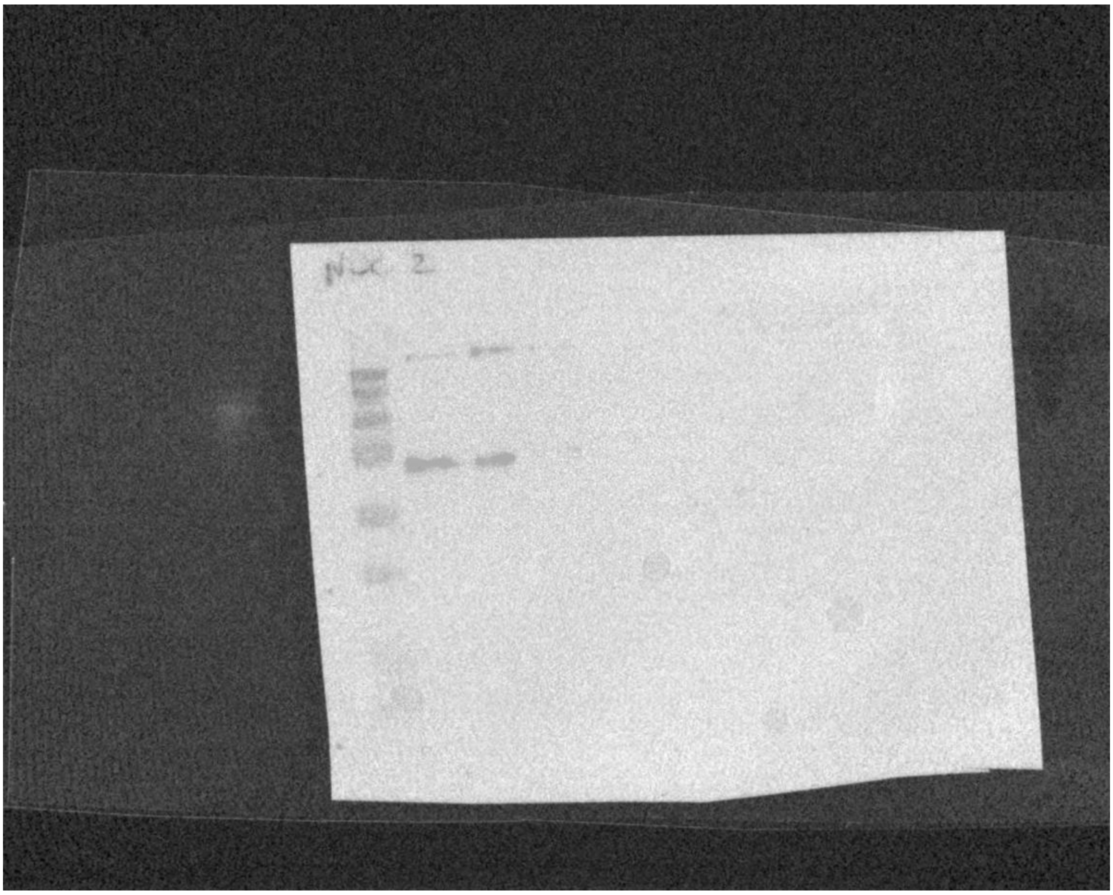

MDA-MB-231_BH
P CREB
Lane 2: Control, Lane 3: IL1β treated

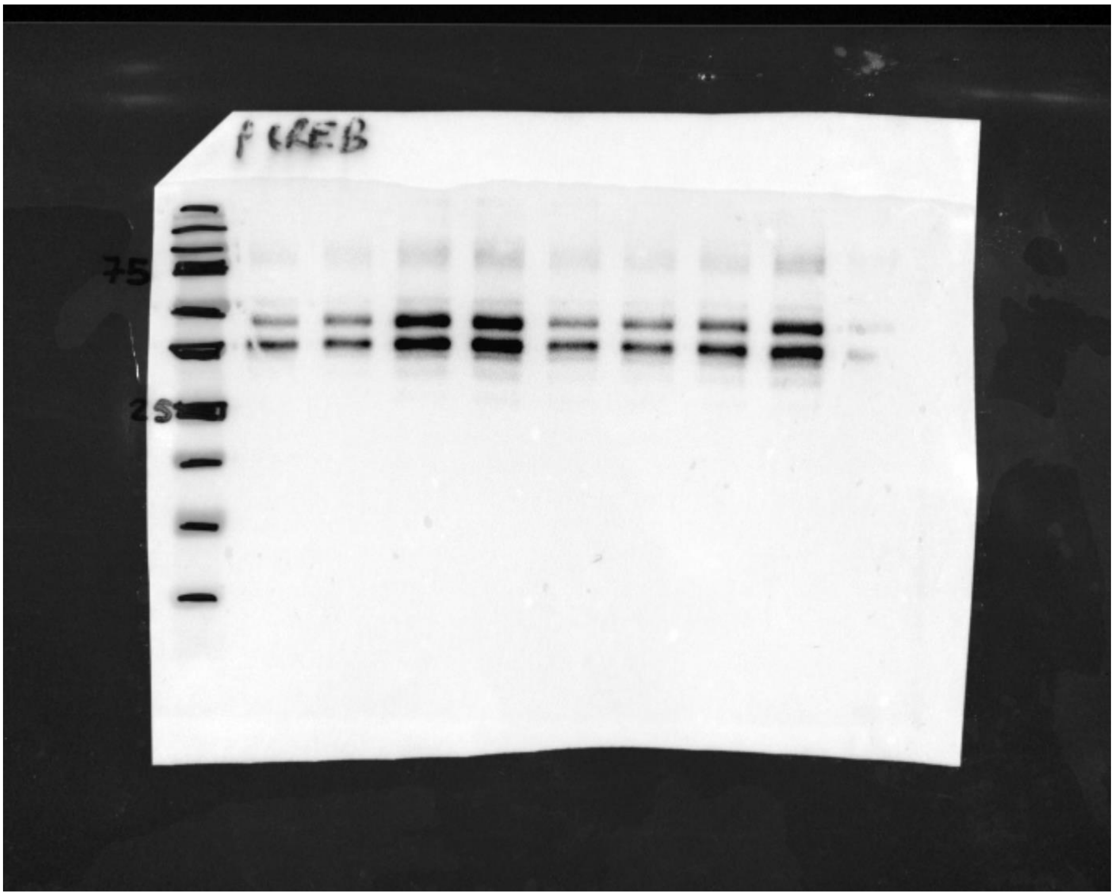

MDA-MB-231_BH
Actin
Lane 2: Control, Lane 3: IL1β treated

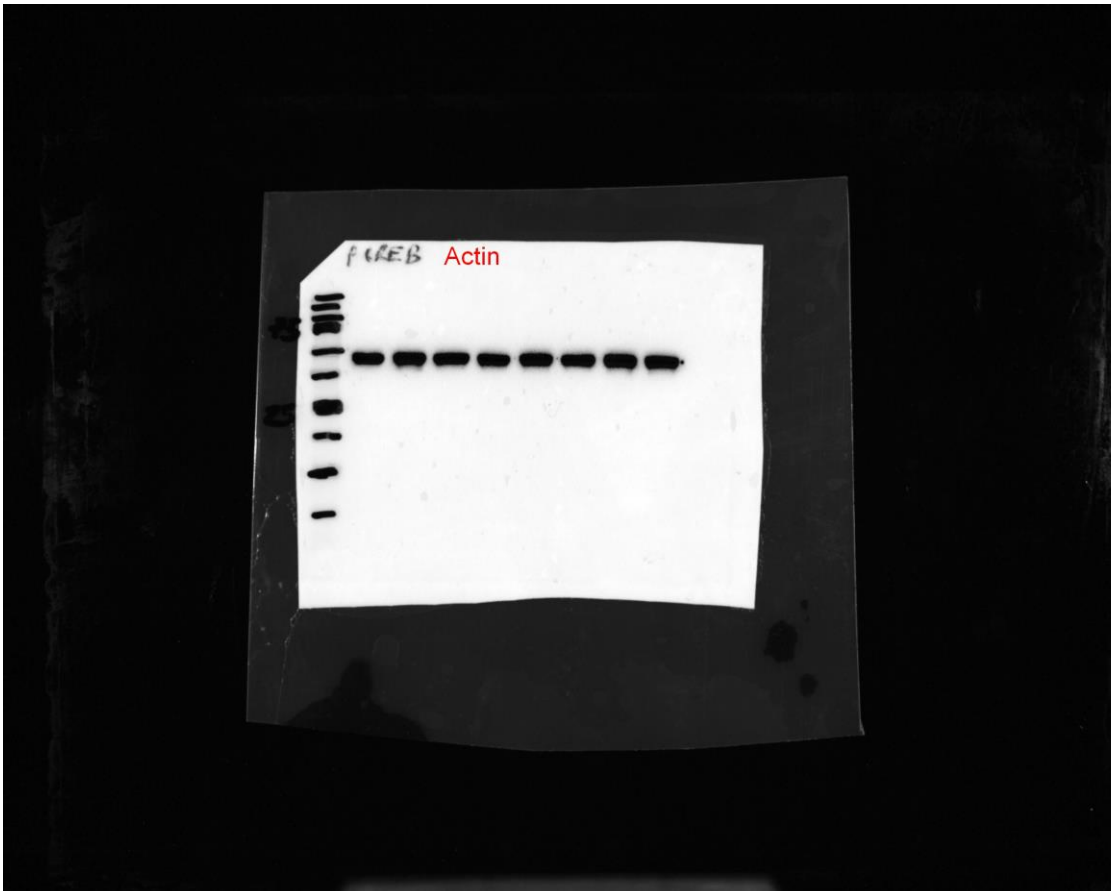

MCF7
P CREB
Lane 1: Control, Lane 2: IL1β treated

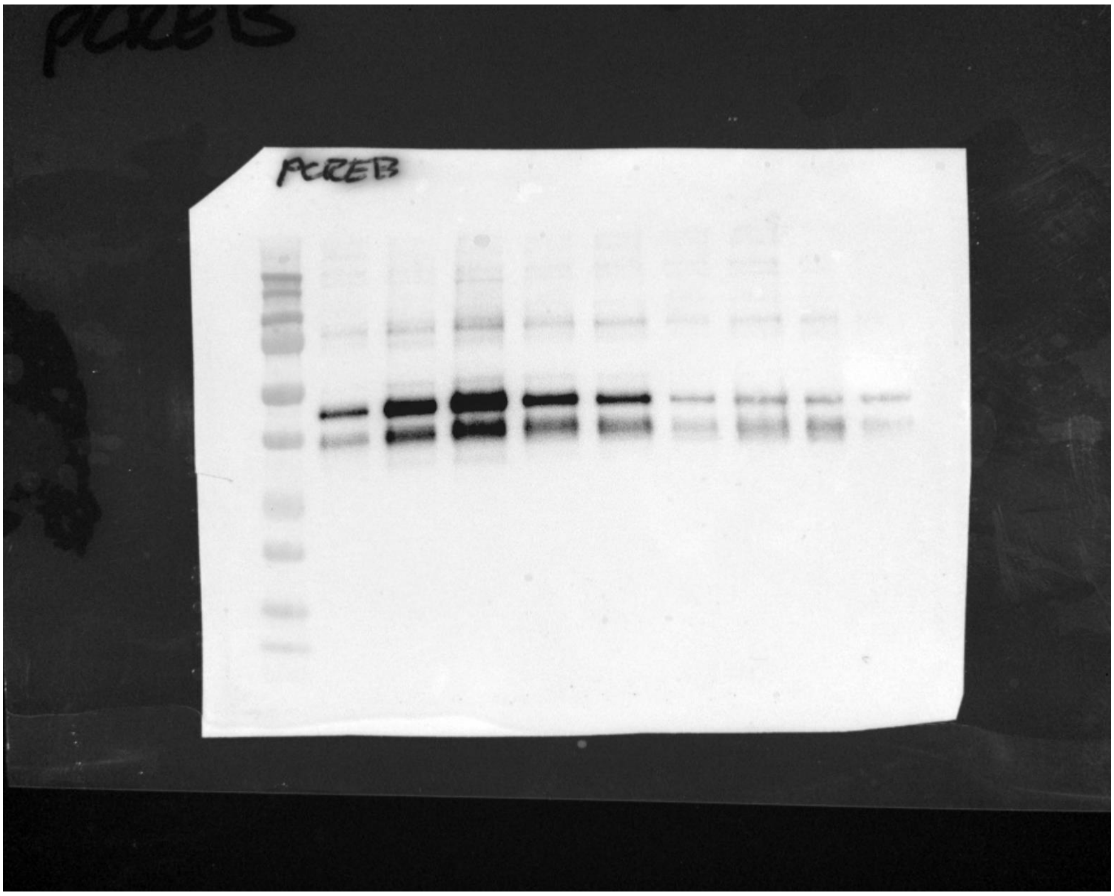

MCF7

Actin

Lane 1: Control, Lane 2: IL1β treated

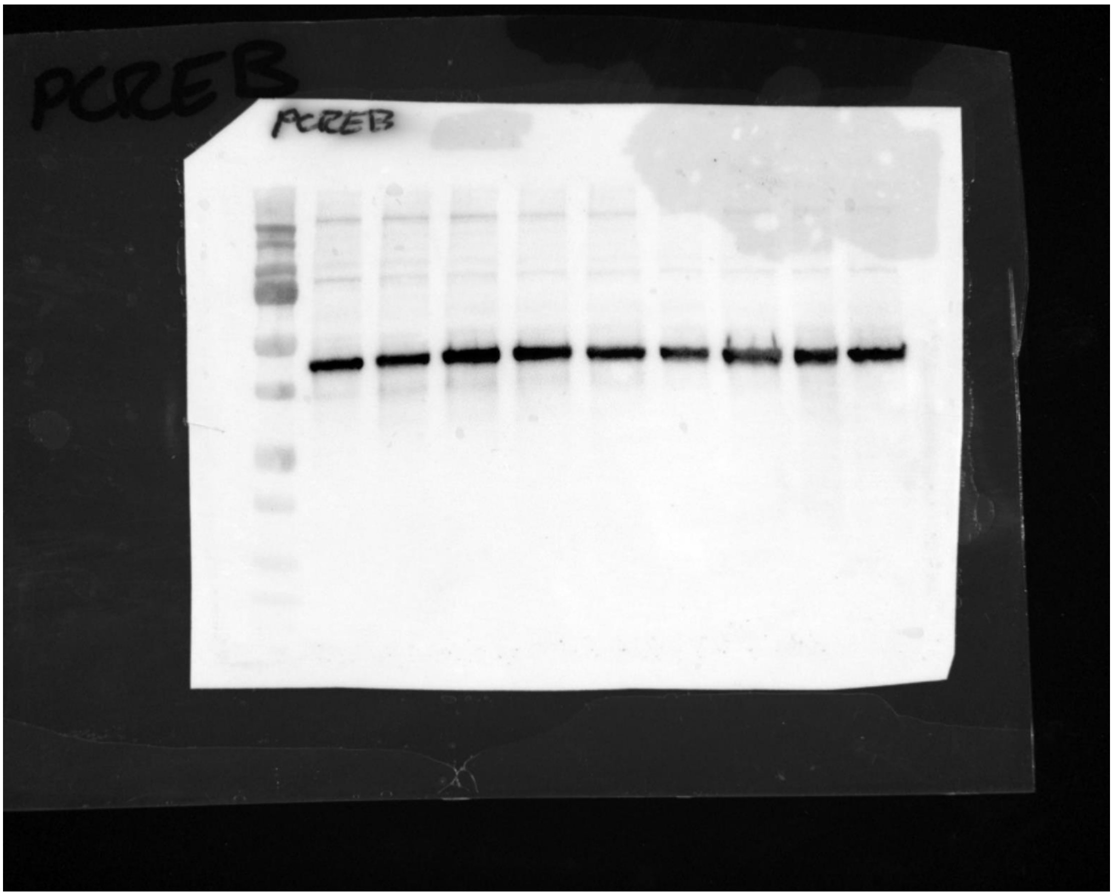

Supplement: Supplementary file 4 — Source Data [file 41467_2019_12807_MOESM4_ESM.pdf]
